# Role of Indwelling Pleural Catheters in Recurrent Exudative Pleural Effusions and Liver Cysts: A Case Report

**DOI:** 10.7759/cureus.62058

**Published:** 2024-06-10

**Authors:** Wei Chee Lee, QinHao Jonathan Ye, Kiran Sharma

**Affiliations:** 1 General Medicine, Sengkang General Hospital, Singapore, SGP

**Keywords:** recurrent effusion, indwelling pleural catheter, large hepatic cyst, exudative pleural effusion, rare cause of pleural effusion

## Abstract

Unilateral exudative pleural effusions have been described as a rare complication of polycystic liver disease. Surgical debridement of the main cyst reduces recurrence of the pleural effusion. We describe the case of an elderly Asian woman with recurrent large right-sided pleural effusion and also a large hepatic cyst under her right hemidiaphragm. She was deemed a poor surgical candidate and was treated with an indwelling pleural catheter (IPC). She was discharged from Sengkang General Hospital with improvement in symptoms.

An 88-year-old Asian woman presented twice to Sengkang General Hospital with recurrent right-sided exudative pleural effusion. She had a past medical history of hypertension, type 2 diabetes, hyperlipidemia, ischemic heart disease (left ventricle ejection fraction 55%), atrial fibrillation, and chronic kidney disease stage 3 (estimated glomerular filtration rate 53). She denied any family history of polycystic kidney or liver disease. Computer tomography of her chest, abdomen, and pelvis revealed a large right pleural effusion and also a large hepatic cyst. A pleural catheter was inserted and the fluid analysis was consistent with an exudative effusion. The pleural fluid was sterile to culture for bacteria and mycobacterium. The cytology was negative for malignant cells. The pleural effusion recurred quickly despite repeated large-volume drainage from the pleural catheter. Our patient was not suitable for surgical debridement of the hepatic cyst and eventually received an IPC and was discharged.

With the advent of IPC, there has been increasing interest in using IPC in the management of non-malignant pleural effusions. While surgical debridement of hepatic cysts is the preferred treatment option in recurrent pleural effusion associated with polycystic liver disease, IPCs now provide another viable and minimally invasive option for clinicians and patients.

## Introduction

Pleural effusion is a common clinical manifestation of multiple conditions, such as diseased pleura, lung, or even systemic conditions or drug-related causes [1]. Rarely, recurrent unilateral pleural effusions as a result of polycystic liver disease have been described in case reports. Surgical intervention in the form of unroofing the major hepatic cyst or hepatic resection has been the treatment of choice in preventing the occurrence of these effusions [2-4]. However, for patients who are poor surgical candidates or have declined surgery, an indwelling pleural catheter (IPC) is an option to prevent the recurrence of the effusion. To the best of our knowledge, we describe the first case of the use of an IPC in an elderly non-surgical patient with recurrent right-sided exudative pleural effusion and a large hepatic cyst.

## Case presentation

An 88-year-old Asian woman presented to Sengkang General Hospital with acute onset of dyspnea for a two-day period. She denied any infective symptoms of cough, sputum production, or fever. No systemic symptoms of weight loss, night sweats, or other cardiac, gastrointestinal, or neurological symptoms were reported.

Significant past medical history included ischemic heart disease (left ventricle ejection fraction 55%), atrial fibrillation, and chronic kidney disease stage 3. She had no previous exposure to asbestos, animals, or farm environment. She had no history of sick contacts, recent illness, travel, or trauma. She did not report any family history of polycystic kidney or liver disease. Other than her chronic medications prescribed by her regular physicians, she did not drink any alcohol, smoke cigarettes, consume leisure or illicit drugs, nor any traditional medications or health supplements.

**Figure 1:**
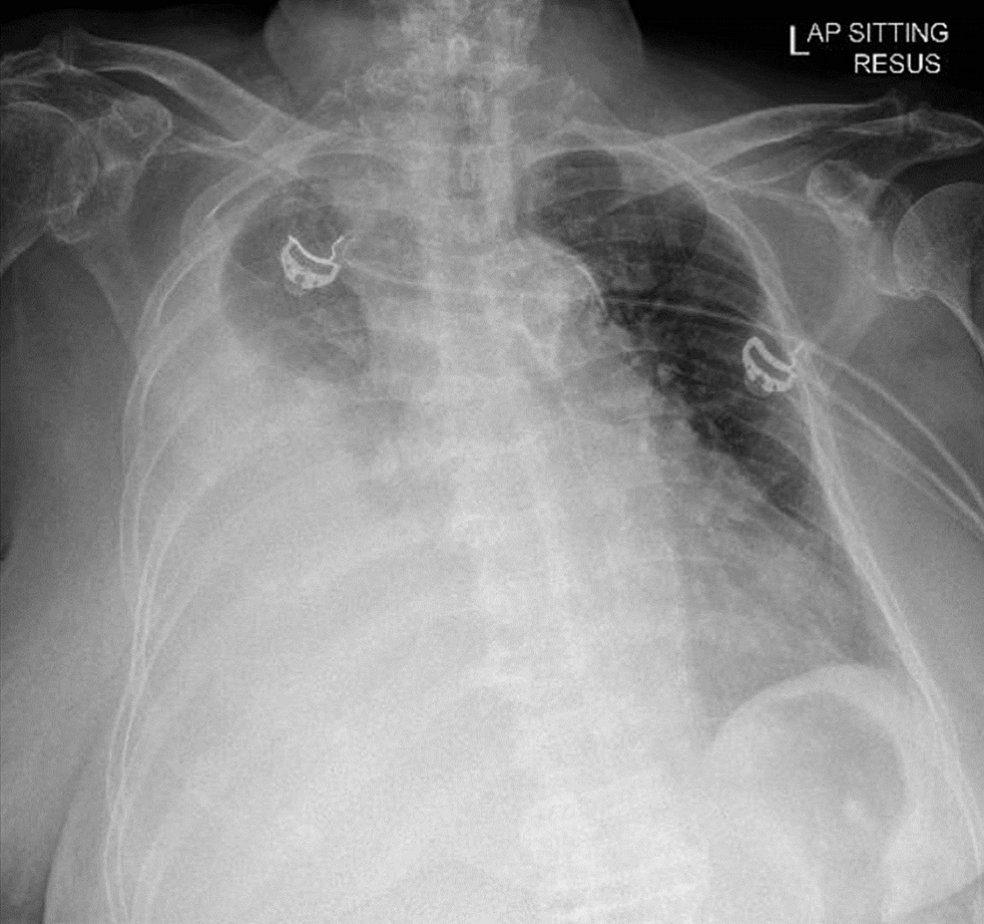
Chest radiograph revealing a large right pleural effusion.

Physical examination revealed poor air entry on her right chest and hepatomegaly. There was also an irregular pulse, but no other abnormalities were found. A chest radiograph confirmed a large right pleural effusion (Figure 1).

**Figure 2:**
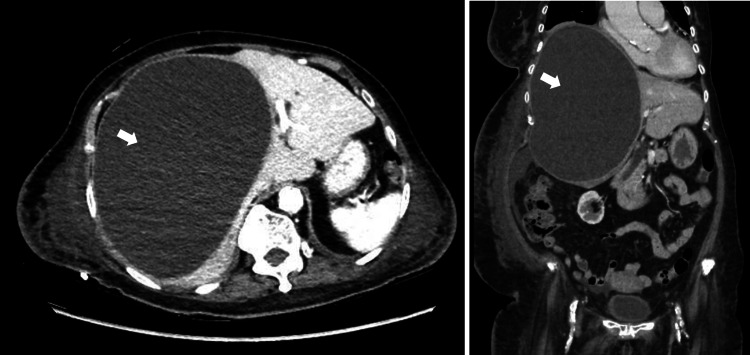
CT scan of abdomen and pelvis (transverse and coronal views) revealing a polycystic liver with a large hepatic cyst measuring 18.9 cm x 13.6 cm x 21.0 cm (white arrows).

Computer tomography (CT) of her chest, abdomen, and pelvis was performed. There was a large right pleural effusion with underlying lung atelectasis. There was no lung or pleural mass seen. No significantly enlarged mediastinal, hilar, axillary, or supraclavicular lymph node was detected. There was also a large cystic lesion, measuring 18.9 x 13.6 x 21.0 cm (Figure 2) and occupying the right lobe of the liver with associated mass effect causing displacement of the right hemidiaphragm superiorly and distortion of the liver architecture and attenuation of the right portal and hepatic veins. There were also bilateral renal cysts seen.

Thoracentesis was performed and the pleural effusion was confirmed to be exudative (protein 30 g/L and lactate dehydrogenase 361U/L) with normal pH (>8), glucose (7.3 mmol/L). Microscopy revealed no bacteria, acid-fast bacilli, or fungi, and cultures were sterile for bacteria, mycobacterium, or fungi. No malignant cells were noted on cytology and BRCA1-associated protein-1 (BAP-1) was tested negative. The pleural fluid analysis did not indicate the cause to be due to infection, pancreatitis, autoimmune disorder, or malignancy. Our patient was discharged after draining 4200 ml of pleural fluid.

However, she was admitted again 16 days later for dyspnea and recurrence of right-sided pleural effusion. Our patient received 14 days of piperacillin and tazobactam. Thoracentesis was repeated and the pleural fluid analysis revealed similar results. The main hepatic cyst was also drained under ultrasound guidance by the interventional radiologist. The microscopy did not reveal any ova, cyst, trophozoite, or parasites. The cultures were sterile for bacteria, mycobacterium, or fungi. No malignant cells were noted on cytology. The appearance of the hepatic cyst fluid was grey and turbid, while the pleural fluid was haemoserous in nature. This suggested that there was no direct communication between the main liver cyst and the pleural cavity.

Despite draining 3650 ml of fluid via the hepatic cyst catheter over a 10-day period, the right-sided pleural effusion continued to reaccumulate. General surgery was consulted for surgical unroofing of the main hepatic cyst. However, in view of her advanced age and pre-existing co-morbidities, our patient was deemed unsuitable for surgery. Thoracoscopy was not performed as the patient and family declined. Due to the rapid recurrence of the right-side pleural effusion, our patient eventually underwent a tunneled PleurX catheter (Denver Biomedical, Golden, CO, USA) insertion after all disease-specific treatments were exhausted and with the intention of providing adequate symptom control with no need for repeated interventions.

Our patient was discharged and reviewed 17 days later in the clinic. Her symptoms remained well-controlled and the drainage volume was reported to be about 200 to 600 ml per day. There were no further admissions related to the pleural effusion or complications of the IPC during a follow-up period of three months.

## Discussion

Exudative pleural effusions have been described as a rare complication of adult polycystic liver disease [5]. Several mechanisms have been postulated for the development of hepatic hydrothorax in liver cirrhosis [6]. Portal hypertension and splanchnic vasodilation lead to the formation of ascites. Due to the negative intrathoracic pressure, there is direct movement of peritoneal fluid into the pleural cavity through diaphragmatic defects. While the underlying mechanism of hepatic hydrothorax is well described in the literature, the pathophysiology of recurrent exudative pleural effusion in polycystic liver disease remains unknown [2].

We found two cases in the literature of large pleural effusions associated with polycystic liver and/or polycystic kidney disease. Woolnough et al. described a 50-year-old Caucasian woman with symptomatic right-sided exudative pleural effusion which was refractory to drainage and only resolved with surgical debridement of the cystic liver [2]. This leads the author to postulate that the pleural effusion occurred as a direct consequence of the mass effect of the main hepatic cyst displacing and deforming the right hemidiaphragm, which in turn led to a disruption of the local capillary permeability resulting in a persistent exudative effusion. Soota et al. described a 48-year-old woman with polycystic kidney and liver disease [3]. She developed recurrent right-sided exudative pleural effusions and was referred for a liver transplant. Surgery was eventually deemed to be high risk and was not recommended in a multi-disciplinary meeting. She was managed expectantly.

To the best of our knowledge, this is the first reported case of an elderly Asian patient with recurrent exudative pleural effusion due to liver cysts. Analysis of the pleural fluid excluded causes of bacterial and mycobacterium infections, malignancy, or rare causes including autoimmune disorder, asbestos-related lung disease, and pancreatitis. Similar to the two previous cases, her pleural effusion was refractory to drainage and recurred rapidly with worsening dyspnea. Our 88-year-old patient had multiple co-morbidities and she declined surgical debridement of the liver cyst. The decision to insert an IPC was made after a balanced discussion with the patient and her family. After the IPC insertion, our patient had not been re-admitted for dyspnea or symptoms related to re-accumulation of the pleural fluid. 

With the advent of IPCs over 15 years ago, there has been increasing interest in their use in the management of non-malignant pleural effusions of various etiologies. 

Yuvarajan et al. published a retrospective analysis of 30 patients with hepatic hydrothorax who received IPCs [7]. Spontaneous pleurodesis was achieved in 18 patients (60%) and IPCs were removed in these patients. The study demonstrated the ability of IPCs to achieve spontaneous pleurodesis in hepatic hydrothorax.

Bhatnagar et al. published a multicentre review of 57 patients and demonstrated the increasing use of IPCs in non-malignant recurrent effusions [8]. Drains were inserted for a variety of indications, which included hepatic hydrothorax, inflammatory pleuritis, empyema, cardiac failure, yellow nail syndrome, and chylothorax. While the rates of complications and pleural infections in these patients were comparable to previous studies on the malignant pleural effusion population, the rate of spontaneous pleurodesis was lower. The authors recommended that IPCs be inserted in select cases when maximal medical therapy has failed to control the symptomatic recurrent pleural effusions.

While surgical therapy is the mainstay of treatment in symptomatic recurrent pleural effusion associated with polycystic liver disease [4], IPCs now provide another viable and minimally invasive option for clinicians and patients. Further studies are still required to clarify the role of IPC in this setting.

## Conclusions

We present a rare case of recurrent exudative pleural effusion in an Asian patient with a large hepatic cyst. While the pathophysiology of hepatic hydrothorax is well described in the literature, the reason for recurrent exudative pleural effusion with a large hepatic cyst remains unknown. It is postulated that the pleural effusion occurred as a direct consequence of the mass effect of the hepatic cyst displacing and deforming the right hemidiaphragm, which in turn led to a disruption of the local capillary permeability resulting in a persistent exudative effusion.

In some reported cases, surgical intervention in the form of unroofing the major hepatic cyst or hepatic resection has been the treatment of choice in preventing the recurrence of these effusions. However, in certain subgroups of patients such as the elderly or those with multiple co-morbidities, surgery may be deemed unsuitable due to high peri-operative risks. Rather than expectant management, indwelling pleural catheter can provide a minimally invasive option for clinicians and patients to reduce the recurrence of pleural effusion and improve symptoms.
